# Biogeographic distributions of microbial communities associated with anaerobic methane oxidation in the surface sediments of deep-sea cold seeps in the South China Sea

**DOI:** 10.3389/fmicb.2022.1060206

**Published:** 2022-12-23

**Authors:** Qiuyun Jiang, Hongmei Jing, Hao Liu, Mengran Du

**Affiliations:** ^1^CAS Key Laboratory for Experimental Study Under Deep-sea Extreme Conditions, Institute of Deep-sea Science and Engineering, Chinese Academy of Sciences, Sanya, China; ^2^University of Chinese Academy of Sciences, Beijing, China; ^3^HKUST-CAS Sanya Joint Laboratory of Marine Science Research, Chinese Academy of Sciences, Sanya, China; ^4^Southern Marine Science and Engineering Guangdong Laboratory (Zhuhai), Zhuhai, China

**Keywords:** cold seeps, anaerobic methane oxidation, 16S rRNA, *mcr*A, *pmo*A, *dsr*B

## Abstract

Cold seeps are oasis for the microbes in the deep-sea ecosystems, and various cold seeps are located along the northern slope of the South China Sea (SCS). However, by far most microbial ecological studies were limited to specific cold seep in the SCS, and lack of comparison between different regions. Here, the surface sediments (0–4 cm) from the Site F/Haima cold seeps and the Xisha trough in the SCS were used to elucidate the biogeography of microbial communities, with particular interest in the typical functional groups involved in the anaerobic oxidation of methane (AOM) process. Distinct microbial clusters corresponding to the three sampling regions were formed, and significantly higher gene abundance of functional groups were present in the cold seeps than the trough. This biogeographical distribution could be explained by the geochemical characteristics of sediments, such as total nitrogen (TN), total phosphorus (TP), nitrate (NO_3_^−^), total sulfur (TS) and carbon to nitrogen ratios (C/N). Phylogenetic analysis demonstrated that *mcr*A and *pmo*A genotypes were closely affiliated with those from wetland and mangroves, where denitrifying anaerobic methane oxidation (DAMO) process frequently occurred; and highly diversified *dsr*B genotypes were revealed as well. In addition, significantly higher relative abundance of NC10 group was found in the Xisha trough, suggesting that nitrite-dependent DAMO (N-DAMO) process was more important in the hydrate-bearing trough, although its potential ecological contribution to AOM deserves further investigation. Our study also further demonstrated the necessity of combining functional genes and 16S rRNA gene to obtain a comprehensive picture of the population shifts of natural microbial communities among different oceanic regions.

## 1. Introduction

Methane is a powerful greenhouse gas with a greater influence than CO_2_, and contributes approximately 22% to the global warming (Dean et al., 2018). Marine sediment is the largest reservoir of methane containing approximately thousands of gigatons (10^15^ g) of carbon stored beneath the seafloor as marine methane hydrates. As one of the largest readily exchangeable carbon reservoirs near Earth’s surface1 (Ruppel and Kessler, 2017), however it contributes only about 2% of the annual global flux of methane to the atmosphere (Cicerone and Oremland, 1988; Ruppel and Kessler, 2017). This is largely due to anaerobic oxidation of methane (AOM), which consumes nearly 90% upward diffusing methane before it was released into the hydrosphere and atmosphere (Hinrichs et al., 2000) and is critical for controlling the methane flux. By far, sulfate-dependent anaerobic methane oxidation (SAMO), denitrifying anaerobic methane oxidation (DAMO) and manganese- and iron-dependent methane anaerobic oxidation are the types of AOM processes found in marine environments (Boetius et al., 2000; Beal et al., 2009).

SAMO is by far the most studied AOM process in marine sediments (Martens and Berner, 1974), and catalyzed by a consortium of anaerobic methanotrophic archaea (ANME-1/2/3) and sulfate-reducing bacteria (SRB) of the genera *Desulfosarcina*/*Desulfococcus* or *Desulfobulbus* (Lösekann et al., 2007). More recently, bacterium Candidatus ‘*Methylomirabilis oxyfera*’ (‘*M. oxyfera*’, NC10) coupling AOM to nitrite reduction through an intra-aerobic methane oxidation pathway (N-DAMO; Ettwig et al., 2010) and a novel ANME lineage named Candidatus ‘*Methanoperedens nitroreducens*’ (ANME-2d) population performing nitrate driven AOM pathway (Nr-DAMO; Hu et al., 2009; Haroon et al., 2013) involved in the DAMO process were reported. Those DAMO-associated groups have been identified in the sediments along the continental shelf and cold seeps of the South China Sea (SCS; Chen et al., 2014; Jing et al., 2020), and were proposed to represent a major methane sink (Jing et al., 2020). The relative importance of DAMO and SAMO seems varied with different marine regions, depending on the types and availability of substrates, and their ecological contributions to the AOM process still need further investigations (Roalkvam et al., 2011; Jing et al., 2020).

Cold seeps are formed by the expulsion of subsurface fluid into the seabed (Paull et al., 1984), and rich in methane and hydrogen sulfide (Jørgensen and Boetius, 2007). Various cold seep sites have been detected on the northern slope of the SCS. Among them, only Haima and Site F are currently active (Feng and Chen, 2015). The Xisha trough is located in the BSR (Bottom-simulating reflectors, geophysical indicator for gas hydrate) area and is gas hydrate-bearing areas of the northern SCS, though no cold seeps are formed currently (Zhang et al., 2019). Based on the 16S rRNA gene, the microbial communities, especially ANME, have been investigated with high-throughput sequencing in Haima (Niu et al., 2017; Zhang et al., 2020) and Site F (Cui et al., 2019). As for the SAMO-associated functional groups, ANME, methanogens and/or SRB have been reported in Haima (Jing et al., 2020; Xu et al., 2021; Guan et al., 2022) and Site F (Feng and Chen, 2015; Zhang et al., 2017; Du et al., 2018; Li et al., 2021). In addition, microbial groups involved in DAMO process were also identified in the cold seeps and the Xisha trough in the SCS (Chen et al., 2014; Jing et al., 2020). However, by far all the molecular microbial ecological studies have been limited to one specific cold seep in the SCS, thus lack of comparisons among different regions, particularly considering different functional groups as a whole.

In the present study, we collected sediment samples from deep-sea cold seeps and the Xisha trough in the SCS to investigate the biogeography of microbial communities, with particular interest in the representative functional groups, e.g., the ANME-2d subcluster, NC10 bacteria and SRB. This study could be better elucidated the spatial variation of the microbial community structure, diversity and associated impacting factors among different regions.

## 2. Materials and methods

### 2.1. Sample collection

**Figure 1:**
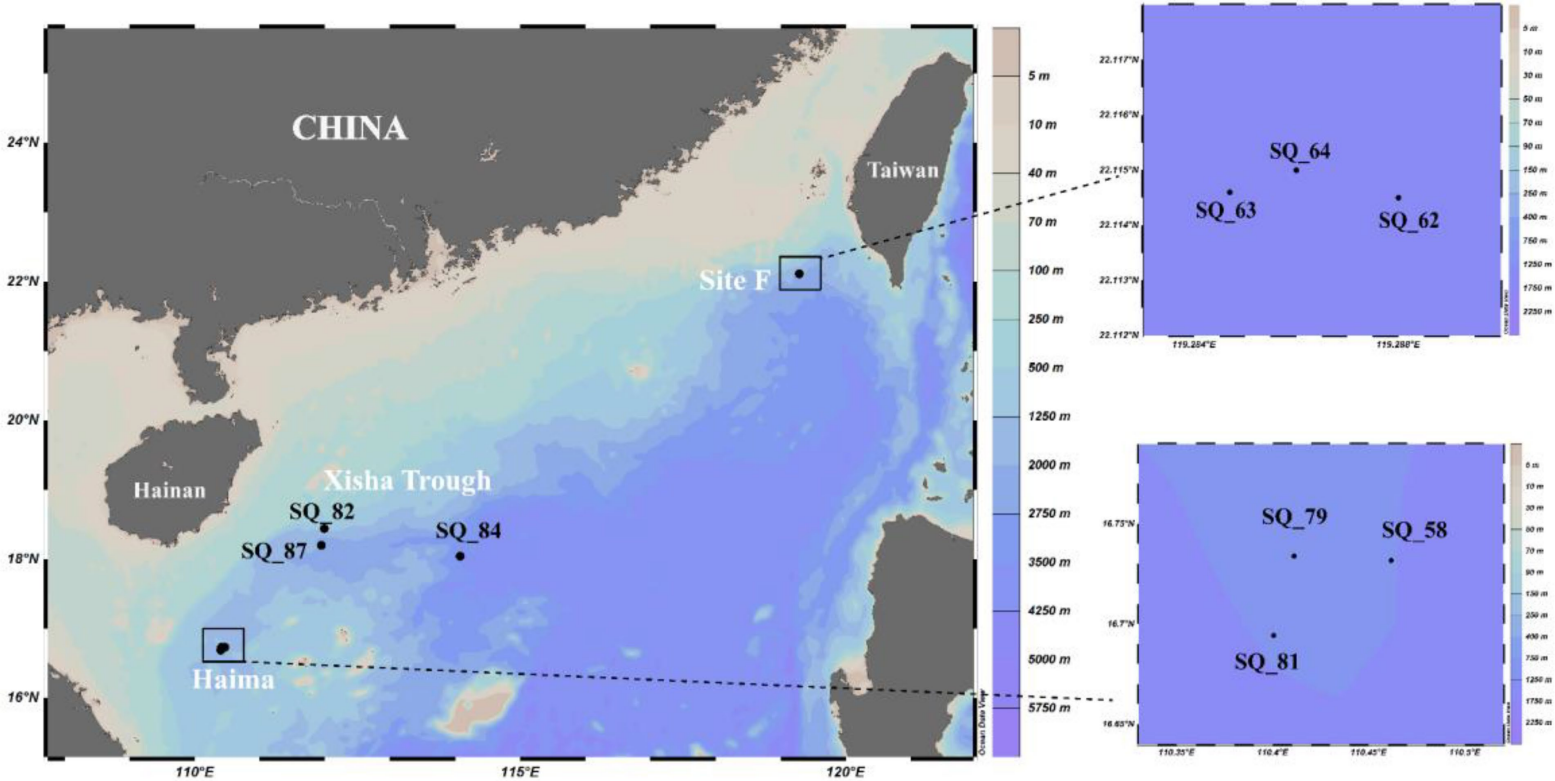
Location of the sampling stations in the South China Sea.

Pushcore sediment samples were collected from two cold seeps (Haima: 16°43’N, 110°28′E; Site F: 22°6’N, 119°17′E) and the Xisha Trough (18°18’N, 114°08′E) located on the continent shelf of the SCS (Figure 1) during cruise TS07 by R/V “Tan Suo Yi Hao” in June 2018 as described previously (Jing et al., 2020). In total, nine stations were sampled, as follows: SQ_54, SQ_79 and SQ_81 (from Haima), SQ_62, SQ_63, and SQ_64 (from Site F), and SQ_82, SQ_84 and SQ_87 (from the Xisha Trough). The surface sediments around 0–4 cm were sliced and then immediately stored at −80°C until further analysis. *In situ* hydrographical parameters (i.e., temperature, depth and location) were recorded during sampling using the manned submersible, SHENHAI YONGSHI.

### 2.2. Chemical analysis of the sediments

Chemical parameters of sediments, including total carbon (TC), total nitrogen (TN), total phosphate (TP), total sulfur (TS), nitrate, and ammonia of each site, were measured at the Institute of Mountain Hazards and Environment, Chinese Academy of Sciences (Chengdu, Sichuan, China), according to Wang et al. (2016). In total, approximately 5 g sediment was used for chemical analysis. Briefly, nitrate and ammonia were detected with a colorimetric auto-analyzer (SEAL Analytical AutoAnalyzer 3, Germany) after 2 M KCl treatment and double qualitative filter paper. TC and TN were determined by over drying the sediments at 105°C and then using an element analyzer (Elementar vario Macro cube, Germany). TS were measured by an inductively coupled plasma-optical emission spectroscopy (ICP-OES; PerkinElmer Optima 8,300, United States). TP was measured with nitric-perchloric acid using the molybdate colorimetric method with a UV2450 (Shimadzu, Japan) after digestion of the sediment (Murphy and Riley, 1962).

### 2.3. DNA extraction and PCR amplification

Triplicate samples at each station were used for DNA extraction and combined for subsequent PCR reaction and sequencing. Genomic DNA in the sediment (~0.5 g) was extracted according to the instruction manual of PowerSoil DNA Isolation Kit (MO BIO Laboratories, Inc., Carlsbad, United States). The extracted DNA was quantified with a NanoDrop 2000 Spectrophotometer (Thermo Scientific, Thermo Fisher Scientific, Corp.) and the quality was checked *via* gel electrophoresis. The DNA samples were stored at −80°C until further processing.

The V3-V4 region of bacterial 16S rRNA gene (468 bp) was amplified by PCR using the primers of 338F (5’-ACTCCTACGGGAGGCAGCAG-3′) and 806R (5′- GGACTACHVGGGTWTCTAAT-3′; Liu et al., 2016), while that of archaeal 16S rRNA gene (462 bp) was amplified with primers of 340F (5’-CCCTAYGGGGYGCASCAG-3′) and 806R (5’-GGACTACVSGGGTATCTAAT-3′; Takai and Horikoshi, 2000). Nested PCR was used for amplification of the *mcr*A gene (363 bp) of *Methanoperedens*-like archaea (ANME-2d; Vaksmaa et al., 2017), the *pmo*A gene (386 bp) of *M. oxyfera*-like bacteria (NC10 bacteria; Luesken et al., 2011), and the *dsr*B gene (350 bp) of *Desulfosarcina*-like sulfate-reducing bacteria (DSRB; Rampinelli et al., 2008). The information of specific PCR primers for above functional genes are listed in Table S1. PCR products were examined with SYBR Safe stained 1.2% agarose gels. The paired-end sequencing of the all amplicons were then performed with an Illumina HiSeq PE250 sequencer (Novogene Co., Ltd.).^1^

### 2.4. Processing and analysis of the sequencing data

To obtain high-quality sequencing data and improve the accuracy of subsequent bioinformation analysis, adapters at both ends of the sequences were removed firstly using the q2-cutadapt plugin after demultiplex; Then, the DADA2 (v1.16) plug-in of QIIME2 v2020.2 was used to filter, dereplicate, identify chimeric sequences, and merge Paired-end (PE) reads (Callahan et al., 2016). PE reads were merged with a minimum length of 12 bp overlap and the representative sequences were picked. The unique amplicon sequence variants (ASVs) table was filtered out by q2-filterfeature after removing the ASVs with frequencies less than 10. The resulting representative ASV sequences for functional genes (*mcr*A, *pmo*A and *dsr*B) were used to calculate phylogenetic trees for subsequent analyses.

Taxonomic classification of 16S rRNA (bacteria and archaea) was processed using the q2-classifysklearn algorithm, and the SILVA (V.132) database was used as a reference with a threshold of 0.8. Annotations were obtained after removing contamination using the q2-feature-table plugin and visualized by the q2-taxa-barplot plugin. The ASVs annotated as mitochondria, chloroplasts, or eukaryotes were also removed using the qiime taxa filter-table and qiime taxa filter-seqs plugins of QIIME2. To detect potential biomarkers, linear discriminant analysis (LDA) effect size (LEfSe) statistical analysis was performed on the Galaxy platform (Segata et al., 2011).^2^ For the prediction of functional and metabolic profiles of the bacterial and archaeal community based on the 16S rRNA gene sequences, the recently developed open-source R package Tax4Fun (Asshauer et al., 2015) was used with the short reads mode disabled along with the SILVA database 123 as required.

Alpha diversity (Shannon and Chao1 indices) and beta diversity were generated with QIIME 2 using q2-diversity, then visualized using box plots and non-metric multidimensional scaling (nMDS) plots at the class level. Pearson’s correlation coefficients were calculated to identify a possible differentiation of the communities under the constraint of environmental factors, and assess correlations between environmental variables and community variability. ANOSIM (analysis of similarities) was used to analyze the similarities of the microbial community compositions among different regions at the class level.

### 2.5. Phylogenetic analysis of functional genes

A phylogenetic analysis of functional gene (*mcr*A, *pmo*A and *dsr*B) sequences was performed using Mega X (Kumar et al., 2018). Reference sequences were retrieved from GenBank database using nucleotide tool BLAST, Popset, and Batch Entrez at NCBI.^3^ The gene sequences were aligned using the ClustalW algorithm of Mega X (Kumar et al., 2018). After testing the best substitution model, phylogenetic trees were constructed using the maximum likelihood method with the substitution model (Tamura-Nei model for *mcr*A, Tamura 3-parameter model for *pmo*A and *dsr*B) with a bootstrap value of 1,000. The phylogenetic tree was visualized and edited through ITOL (V2.0; Letunic and Bork, 2006).

### 2.6. Quantitative PCR

The abundance of the functional genes (*mcr*A, *pmo*A and *dsr*B) was quantified using the Bio-Rad System (Bio-Rad Inc., United States) and TB Green Premix^®^ Ex Taq II (Takara Bio Inc., Shiga, Japan) with primers McrA159F/McrA345R (Vaksmaa et al., 2017), cmo182F/cmo568R (Luesken et al., 2011) and DSRp2060F/DSR4R (Geets et al., 2006) respectively. The specific information for all the qPCR primers were listed in Table S1. Standard curves were constructed using a series of tenfold dilutions of the standard plasmids (known copy number) containing the targeted genes. Triplicate qPCR reactions were performed for each sample with double-distilled water as a negative control, and the gene copy number was normalized to the quantity of the gene.

## 3. Results

### 3.1. Geochemical characterization of the sediments

The Xisha trough was located in between the two seep regions, and geographically closer to Haima (Figure 1). The highest contents of TN (0.09% ~ 0.17%) and TC (0.67% ~ 1.27%) were found in Haima (Table 1). The carbon to nitrogen ratios (C/N; 6.87 ~ 8.30) were higher in the cold seeps than in the trough, and the highest values were found at Stn. SQ64 in Site F. The concentration of NH_4_^+^ (9.49 ~ 17.10 mg/kg) was highest at Stn. SQ64 in the Site F and lowest at Stn. SQ81 in the Haima. The highest and lowest concentration of NO_3_^−^ (1.01 ~ 1.41 mg/kg) was detected at respective Stns. SQ87 and SQ82 in the Xisha trough. TP (477.01 ~ 1,071.21 mg/kg) was significantly higher in Site F, especially at Stn. SQ64. In general, Site F contained high concentrations of TP, NH_4_^+^ and C/N ratio, whereas Haima had high TN and TC contents.

**Table 1:** Geochemical information of sediment samples collected from cold seeps in the South China Sea.

Station		Longitude (°E)	Latitude (°N)	Depth (m)	TN	TC	TP (mg/kg)	TS (mg/kg)	C/N Ratio	NH_4_^+^ (mg/kg)	NO_3_^−^(mg/kg)
					(%)	(%)					
SQ58	Haima	110.46	16.73	1,388	0.17	1.27	678.28	1,468.90	7.66	11.86	1.23
SQ79		110.41	16.73	1,377	0.15	1.15	531.86	3,017.88	7.87	10.46	1.02
SQ81		110.4	16.69	1,366	0.16	1.18	477.01	1,574.94	7.41	9.49	1.05
SQ62	Site F	119.29	22.11	1,151	0.09	0.67	812.62	529.79	7.61	10.05	1.31
SQ63		119.28	22.11	1,165	0.1	0.75	918.97	137.42	7.84	15.84	1.06
SQ64		119.29	22.12	1,305	0.1	0.81	1,071.21	161.85	8.3	17.1	1.12
SQ82	Xisha trough	111.99	18.44	1,732	0.14	1.11	610.99	1,127.57	8	13.11	1.01
SQ84		114.08	18.05	3,408	0.1	0.67	555.34	925.19	6.87	11.63	1.22
SQ87		111.94	18.2	2,200	0.12	0.91	659.45	904.08	7.56	9.8	1.41

### 3.2. Diversity and structure of bacterial and archaeal communities

**Figure 2:**
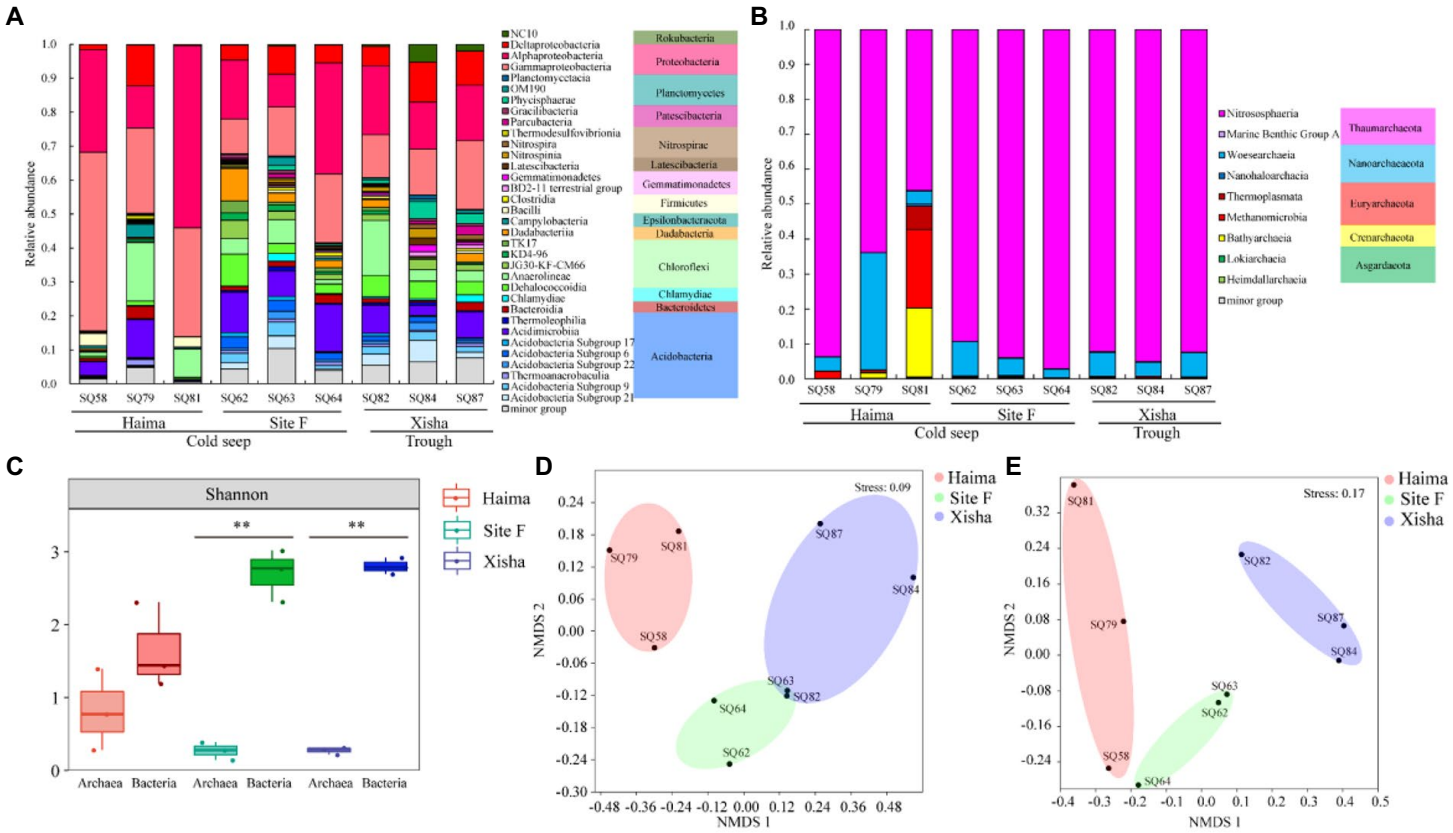
Community composition of bacteria **(A)** and archaea **(B)** at the class level; Averaged alpha diversity indices of the bacterial and archaeal communities based on 16S rRNA gene (**: *p* < 0.01) **(C)**; non-metric multidimensional scaling (NMDS) plots of bacterial **(D)** and archaeal **(E)** communities based on 16S rRNA gene.

The sequencing process yielded 460,109 quality reads for bacterial 16S rRNA gene and 549,475 quality reads for archaeal 16S rRNA gene from 9 stations (Table S2). A total of 9,251 ASVs from bacterial 16S rRNA gene and 5,234 ASVs from archaeal 16S rRNA gene were identified in all nine samples. The bacterial ASVs were classified into 18 phyla and 34 classes, and the archaeal ASVs were classified into 9 phyla and 9 classes (Figures 2A,B). The average alpha diversity (Shannon) of bacterial community at the class level was significantly higher than that of archaeal community in the three regions, especially in the Site F and the Xisha trough (*p* < 0.01, Figure 2C). NMDS analysis of bacteria and archaea at the class level showed that three distinct clusters were formed, corresponding to the three sampling regions (Figures 2D,E).

The major bacterial phyla in all samples were *Proteobacteria*, *Chloroflexi* and *Acidobacteria* (Figure 2A). Among them, *Proteobacteria* was the predominant bacterial phylum, and comprised mainly of α- and γ-*Proteobacteria* (Figure 2A). In addition, NC10 bacteria belonging to *Rokubacteria* were detected in all study areas (Figure 2A). Within the 9 archaeal phyla, *Thaumarchaeota* was the most abundant archaeal phylum in all samples, and dominated by *Nitrososphaeria* (Figure 2B). *Thermoplasmata* and *Methanomicrobia* belonging to *Euryarchaeota,* together with *Bathyarchaeia* (*Crenarchaeota*) occupied a higher proportion in Haima especially at Stn. SQ81 (Figure 2B). The relative abundance of Marine Benthic Group *A*, *Thaumarchaeota* and *Thermoplasmata,* was higher in the trough than the cold seeps, while that of *Methanocellales* and *Methanomicrobia* was higher the in cold seeps than the trough (Figure 2B).

### 3.3. Comparison between the cold seeps and the trough

**Figure 3:**
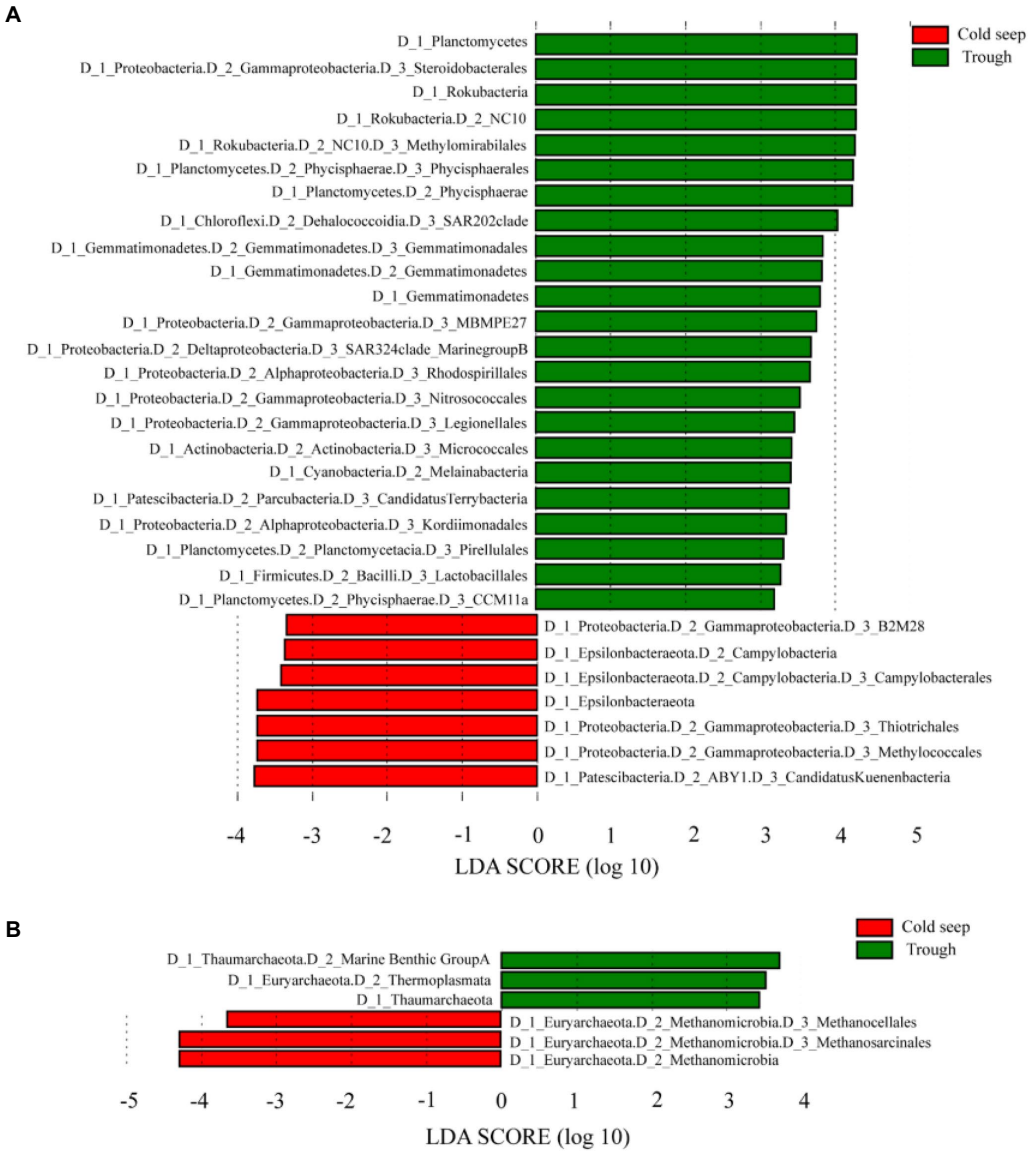
LEfSe analysis between the cold seeps and the trough for bacteria **(A)** and archaea **(B)** showing the Linear Discriminant Analysis (LDA) score.

LEfSe analysis demonstrated that NC10 bacteria (*Rokubacteria*) and *Methylococcales* accounted for higher proportion in the trough and the cold seeps, respectively (*p* < 0.05, Figure 3A; Table S3). The relative abundance of *Marine Benthic Group A*, *Thaumarchaeota* and *Thermoplasmata,* was higher in the trough than in the cold seeps, while that of *Methanocellales* and *Methanosarcinales* showed the opposite trend (*p* < 0.05, Figure 3B).

For functional prediction of bacteria with Tax4fun based on total ASVs of bacterial 16S rRNA gene, the abundance related to acetoclastic methanogens was the highest in all methanogenesis pathways, especially in the Xisha trough, while that related to CO_2_ and methylotrophic methanogens was higher in the cold seeps (Figure S1A). The genes related to denitrification and dissimilatory nitrate reduction was more abundant in the Xisha trough, while that of assimilatory sulfate reduction was more abundant in Site F (Figure S1A).

For functional prediction of archaea with Tax4fun based on total ASVs of archaeal 16S rRNA gene, the abundance of methane metabolism was the highest in the cold seeps especially in Haima, while that of denitrification, dissimilatory nitrate reduction and assimilatory sulfate reduction was higher in the Xisha trough (Figure S1B).

### 3.4. Phylogeny of functional genes

**Figure 4:**
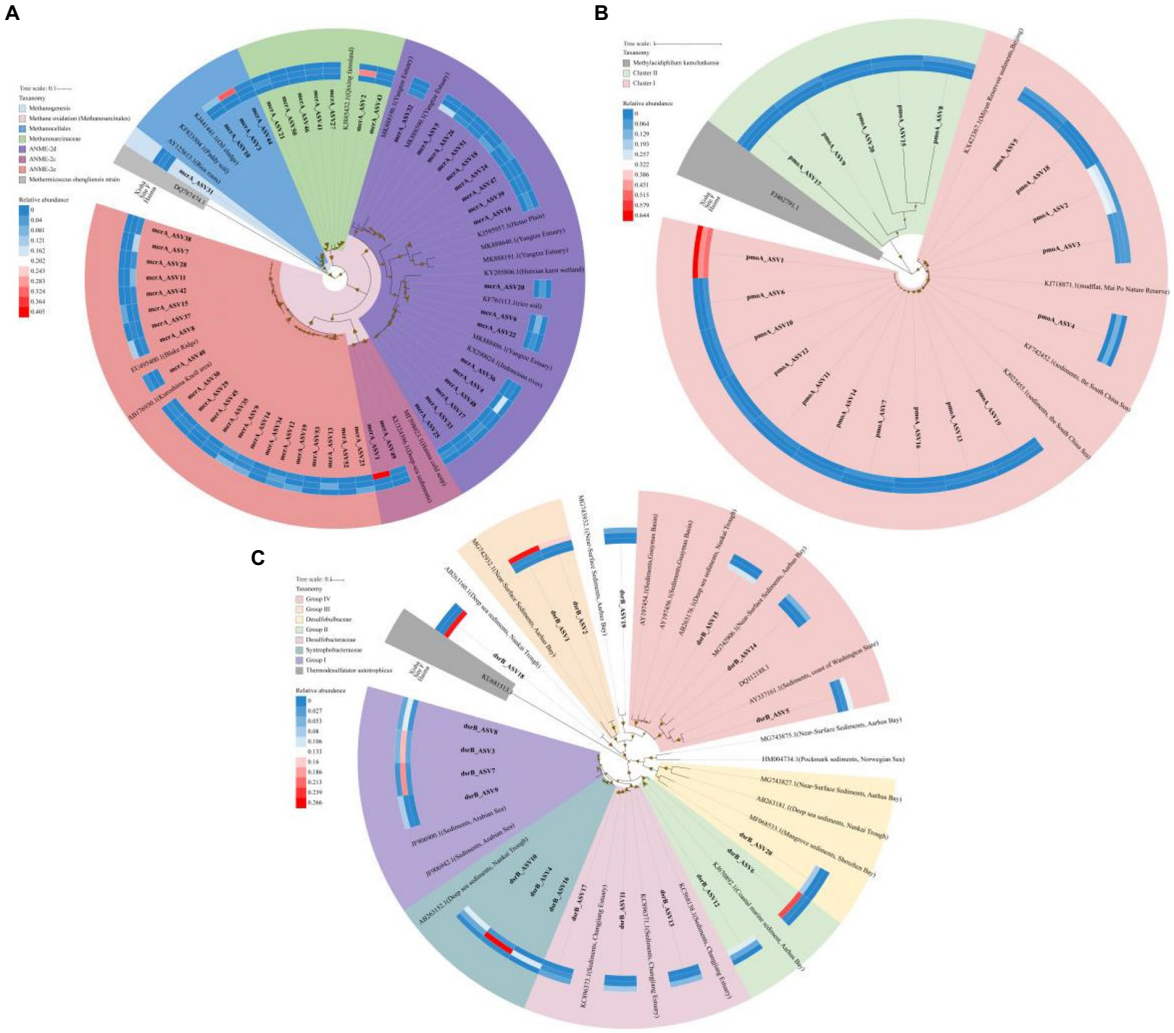
Maximum likelihood phylogenetic trees for *mcr*A gene with top 53 ASVs (accounting for >95% of the total retrieved *mcr*A gene sequences) **(A)**, *pmo*A gene of top 20 ASVs (accounting for >85% of the total retrieved *pmo*A gene sequences) **(B)** and *dsr*B gene of top 20 ASVs (accounting for >49% of the total retrieved *dsr*B gene sequences) **(C)**. Bootstrap values over 50% based on 1,000 replicates were shown. The abundance of the ASVs in each region was shown in the heatmap referred to the color key.

A total of 638,497 high-quality *mcr*A gene sequences were obtained from all samples excluding Stn. SQ81, and were classified into 222 ASVs using DADA2 (Table S2). Top 53 ASVs (accounting for >95% of the total retrieved *mcr*A gene sequences) were used to construct a phylogenetic tree, and fell into five distinct clusters, i.e., ANME-2e, ANME-2c, ANME-2d, *Methanosarcinaceae*, and *Methanocellales* (Figure 4A). ANME-2e (22ASVs, 27.9%) and ANME-2d (18ASVs, 27.6%) were the two major clusters.

A total of 624,726 high-quality *pmo*A gene sequences were obtained in all nine samples, and were classified into 251 ASVs using DADA2 (Table S2). Top 20 ASVs (accounting for >85% of the total retrieved *pmo*A gene sequences) were used to construct a phylogenetic tree, with two distinct clusters formed (Figure 4B). Cluster I contained most of the ASVs (15 of 20 ASVs).

A total of 696,892 high-quality *dsr*B gene sequences were obtained in all nine samples and were classified into 2,646 ASVs using DADA2 (Table S2). Seven distinct clusters were formed based on the top 20 *dsr*B ASVs with affiliated sequences from the GeneBank database, i.e., Group I, *Syntrophobacteraceae*, *Desulfobacteraceae*, Group II, *Desulfobulbaceae*, Group III and Group IV (Figure 4C).

### 3.5. Composition, diversity and abundance of functional genes

For *mcr*A gene, the highest proportion of *Methanosarcinaceae* and lowest proportion of ANME-2e were detected in Site F (Figure 4A). ANME-2c and *Methanocellales* were major group in the Haima and the Xisha trough, respectively. The relative abundance of ANME-2d was higher in the cold seeps (accounting for 23.3% in the Haima and 42.4% in the Site F, respectively) than that in the trough (accounting for 4.1%; Figure 4A). Meanwhile, distinct ANME-2d predominant in each sample, e.g., ASV5, ASV4 and ASV24 was mainly distributed in the Haima, the Site F and the Xisha trough, respectively, (Figure S1C). For *pmo*A gene, Cluster I affiliated with NC10 bacteria accounted for higher proportion in the cold seeps than the trough. ASV1 dominant in all samples, while ASV2 was mainly in the cold seeps (Figure S1D). For *dsr*B gene, Group I and *Syntrophobacteraceae* were mainly in Site F (respective of 44.3 and 36.5%); higher relative abundance of *Desulfobacteraceae*, Group III and Group IV were found in the Xisha trough, while Group II and *Desulfobulbaceae* accounted for higher proportion in the Haima (Figure 4C; Figure S1E).

**Figure 5:**
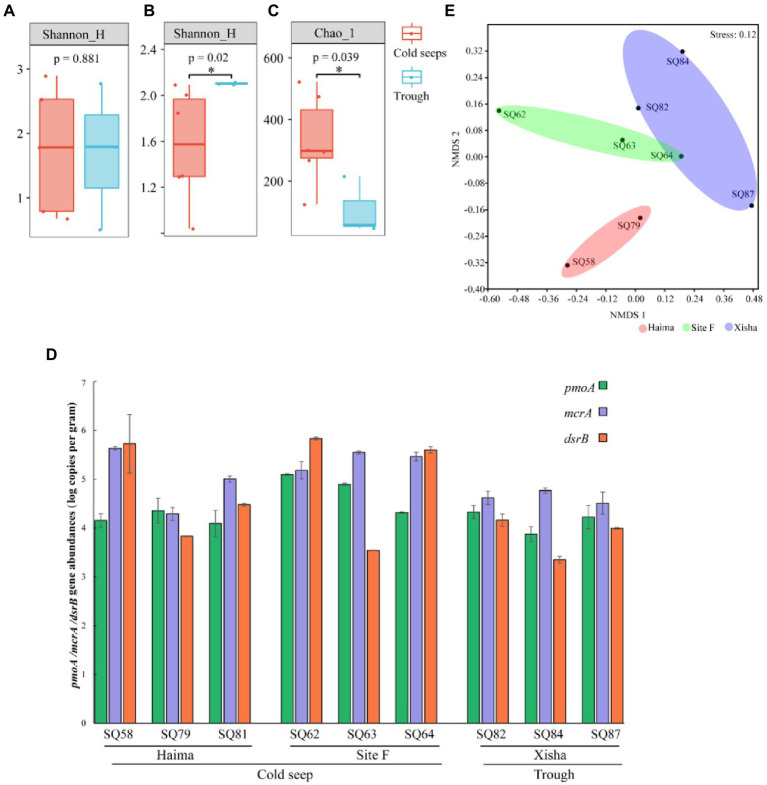
The averaged alpha diversity in different regions for *mcr*A **(A)**, *pmo*A **(B)** and *dsr*B **(C)** genes (*p < 0.01); Gene copy numbers (log copies in wet sediment weight) **(D)** and three functional genesnon-metric multidimensional scaling (NMDS) plot of different functional genes **(E)**.

In terms of alpha diversity, there was no significant difference of *mcr*A group between the cold seeps and the trough (Figure 5A), while significantly higher diversity of *pmo*A and *dsr*B groups was present in the trough and the cold seeps, respectively (*p* < 0.05, Figures 5B,C). Regarding gene abundance quantified by qPCR, significantly higher abundance of *mcr*A, *pmo*A and *dsr*B gene was detected from the cold seeps than from the trough (*p* < 0.05, Figure 5D). NMDS analysis of functional genes showed that three distinct clusters corresponding to the different sampling regions were formed (Figure 5E).

### 3.6. Environmental impacts

Pearson’s correlation coefficients demonstrated that significant correlation existed for Group II/III of *dsr*B with TS and C/N Ratio, and for ANME-2c/2d of *mcr*A with TS and TP (*p* < 0.05, Table 2). Regarding alpha diversity, it was significantly correlated with TN and TC in the bacterial community, and with TN, TC and TS in the archaeal community (*p* < 0.05, Table 2).

**Table 2:** Pearson correlation matrix among the variables.

	Variables	TN	TC	TP	TS	C/N Ratio	NH_4_^+^	NO_3_^−^
*dsr*B	Group I	−0.204	−0.116	0.315	−0.458	0.429	0.412	−0.301
	Syntrophobacteraceae	−0.198	−0.191	0.22	−0.284	0.101	−0.083	−0.088
	Desulfobacteraceae	−0.111	−0.099	0.102	−0.177	0.051	−0.145	0.629
	Group II	0.345	0.398	−0.252	**0.788**	0.267	−0.158	−0.456
	Desulfobulbaceae	0.61	0.59	−0.112	0.303	0.003	−0.089	0.113
	Group III	−0.336	−0.458	−0.238	−0.098	**−0.718**	−0.021	0.148
	Group IV	0.344	0.272	−0.133	0.067	−0.317	−0.15	0.538
*mcr*A	ANME-2e	−0.049	−0.134	−0.238	−0.074	−0.505	−0.161	0.251
	ANME-2c	0.276	0.32	−0.328	**0.816**	0.168	−0.234	−0.359
	ANME-2d	−0.239	−0.129	**0.705**	−0.287	0.62	0.57	−0.033
	Methanosarcinaceae	−0.397	−0.364	0.459	−0.442	0.16	0.517	−0.244
	Methanocellales	−0.003	−0.001	−0.13	−0.085	−0.034	−0.293	0.563
*pmo*A	Cluster I	0.467	0.53	−0.1	0.316	0.397	−0.152	−0.409
	Cluster II	−0.467	−0.53	0.1	−0.316	−0.397	0.152	0.409
Diversity	Shannon (bac)	**−0.789**	**−0.759**	0.325	−0.434	−0.021	0.274	0.221
	Shannon (arc)	**0.767**	**0.738**	−0.527	**0.799**	−0.061	−0.534	−0.23
	Shannon (*mcr*A)	−0.32	−0.344	0.204	−0.261	−0.182	0.097	0.331
	Shannon (*pmo*A)	0.197	0.194	−0.432	0.392	−0.069	−0.241	−0.039
	Chao_1 (*dsr*B)	0.597	0.653	−0.137	0.655	0.366	−0.166	−0.335
Gene abundance	*mcr*A	−0.03	0.008	0.63	−0.429	0.313	0.57	−0.01
	*pmo*A	−0.568	−0.534	0.439	−0.394	0.121	0.025	0.188
	*dsr*B	0.383	0.373	0.122	0.034	0.055	−0.014	0.273

Bold values in the table represent the better positive correlation between the variables (*p* < 0.05).

## 4. Discussion

### 4.1. Heterogeneity of bacterial and archaeal communities

Bacterial community exhibited higher diversity than that of archaeal community in the three studied regions in this study, consisting with the finding of previous studies conducted in the cold seeps of the SCS (Zhang et al., 2012; Cui et al., 2019; Jing et al., 2020). Compared with trough, higher proportions of SRB (*Desulfobulbus*, *Desulfococcus* and *Desulfobacteraceae*) were found in the cold seeps, where typically syntrophic consortium formed by those SRB groups with anaerobic methane oxidizers (Boetius et al., 2000; Orphan et al., 2001). On the other hand, NC10 bacteria accounted for higher proportion in the trough, where has been proposed to support a widespread occurrence of this bacterial group (Chen et al., 2014) with the potential of performing N-DAMO pathway with the co-operation of anammox bacteria (Hu et al., 2009; Ettwig et al., 2010). In addition, *Methylococcales* (type I methanotrophs) occupied a high proportion in the cold seeps, very possibly be favored by relatively high methane released after AOM process (Hanson and Hanson, 1996).

As for archaea, the prevalence of methylotrophic *Methanosarcinales* and acetogenotrophic *Methanocellales* in the cold seep sediments suggested a potentially high level of *in situ* methane production, and in agreement with the functional prediction based on 16S rRNA genes in this study. In addition, archaeal groups, i.e., *Methanoperedenaceae* (ANME-2d), ANME-3, ANME-2a/2b and ANME-2c, were mainly detected in the cold seeps. It was already known that ANME −2a, −2b, −2c, and − 3 could form a consortium with SRB to participate in the SAMO process (Knittel and Boetius, 2009), therefore, their presence might further prove that SAMO processes widely occur in the hydrate-bearing seeps of the northern SCS (Niu et al., 2017; Cui et al., 2019). The detection of ANME-2d clades *Methanoperedens* in the cold seeps indicated that the DAMO-related microbes existed in the hydrate-bearing cold seeps (Haroon et al., 2013), although their real ecological role in this process still need direct proofs. Different ANME clades had varied spatial distribution, for example, ANME-3 predominant in this study, while ANME-1 and ANME-1b predominant in the respective of Jiulong methane reef area (Cui et al., 2016) and the GMGS2 drilling area of the SCS (Cui et al., 2019). This reflected the complexity and heterogeneity of the microbial communities in different cold seeps, and the necessity of conducting specific studies on each individual seep.

### 4.2. Phylogeny and abundance of functional groups

Based on the phylogeny of *mcr*A gene ANME-2d cluster affiliated with sequences recovered from Yangtze Estuary sediment (Zheng et al., 2020), wetland (Chen et al., 2017) and Indonesian river sediment (Vaksmaa et al., 2017), which were typical habitats for the occurrence of methanogenesis (Wang et al., 2015) and Nr-DAMO processes (Haroon et al., 2013). Higher proportion of ANME-2d in the cold seeps was consistent with the findings based on archaeal 16S rRNA gene analysis in this study, although this archaeal cluster was undetectable in the Haima cold seep previously using another conventional *mcr*A primer (Niu et al., 2017). As for *pmo*A gene, two distinct clusters formed, with Cluster I was closely related to sequences recovered from the mudflat in Mai Po Nature Reserve of Hong Kong (Chen et al., 2015a) and sediments of the SCS (Chen et al., 2014, 2015b). NC10 bacteria have been reported using *pmo*A gene from the sediments in the SCS previously (Chen et al., 2014, 2015b, 2022). The association of ANME-2d archaea and NC10 bacteria with DAMO process has been proposed (Chen et al., 2022), future studies with isotopic tracing experiments will be helpful to elucidate their functions and relative contributions to this process.

Comparatively, more distinct clusters were formed for *dsr*B gene, suggesting highly diversified *dsr*B genotypes existed. Among the seven clusters, clusters of *Syntrophobacteraceae, Desulfobulbaceae* and Group IV were closely related to sequences from deep-sea sediments of Nankai Trough (Kaneko et al., 2007); *Desulfobacteraceae* cluster was closely related to sequences from sediments of Changjiang Estuary (He et al., 2015); clusters of Group II/III were closely related to sequences from coastal marine sediment of Aarhus Bay (Petro et al., 2019). The high diversity of SRB genotypes might be associated with the different ANME groups detected in the same region. This phenomenon has also been observed in the pockmark (Lazar et al., 2011) and Thuwal cold seeps (Lee et al., 2014), might be influenced by the sulfate concentrations and organic substrates availability (Leloup et al., 2007; Lazar et al., 2011). Highly diversified substrate utilizing capability of different SRB groups reflected a better adaptability to the deep-sea environment (Dhillon et al., 2003), and a potentially higher ecological contribution to AOM progress in the cold seeps and trough.

Higher gene abundance of all the three functional groups in the cold seeps than in the trough might suggest that those functional groups were selectively favored by the enriched substrates in the cold seeps (Zhou et al., 2015; Niu et al., 2017; Cui et al., 2019). In the Xisha trough, significantly higher abundance of NC10 group might be associated with the frequently predicted functions of denitrification and dissimilatory nitrate reduction. This suggested that N-DAMO process was more important in the hydrate-bearing trough, although its contribution to the AOM process should be estimated using the isotopic tracing and active microbial assemblages involved deserve further investigation at the RNA level.

### 4.3. Impacting parameters on the microbial groups

Distinct prokaryotic and functional microbial communities were formed in the three respective hydrate-bearing locations. Distinct microbial communities formed in different cold seeps have been reported worldwide, and were locally selected by the biotic and abiotic factors (Ruff et al., 2015; Vigneron et al., 2019). The degree of microbial endemism in the methane seep suggests a high local diversification in the heterogeneous cold-seep ecosystems (Ruff et al., 2015). The clear biogeographical distribution pattern of microbial communities were attributed to the *in situ* geochemical conditions in the sediments, especially TN, TC, TP, NO_3_^−^, and TS; and their importance driving the spatial distribution of microbial communities in the cold-seep ecosystems have been reported previously (Heijs et al., 2007; Roalkvam et al., 2011; Shao et al., 2014; Niu et al., 2017). However, by far different environment parameters were measured in different studies, and it is difficult to ascertain which one was the most important factor. Previous studies indicated strong correlations of microbial communities in the cold seep sediment with the concentrations (Zhang et al., 2012) and physical forms of methane (Cui et al., 2019). In those hydrate-bearing ecosystems, it would be reasonable to assume that the concentrations and physical forms of methane as the key drivers, although no *in situ* methane concentration was reported in most related studies. Methane concentrations were not measured in our study, but bubbling of fluids from the cold seeps were observed during diving with the deep-sea HOV in the cruise. Haima has exhibited a decline in activity in recent years (Liang et al., 2017), while Site F is currently active (Feng and Chen, 2015) with high concentration of methane gas (Lin et al., 2007), and the Xisha trough with large amount of gas hydrate, without cold seeps formed currently (Zhang et al., 2019). Therefore, concentrations and physical forms of methane in those different regions would be different, subsequently supporting distinct prokaryotic and functional communities, and should be measured and recorded in the future studies.

### 4.4. Necessity of functional genes

16S rRNA genes as phylogenetic markers for amplicon sequencing have become routine in the microbial ecology studies in recent years (Woese and Fox, 1977), but functional genes that are indicative of a particular microbial guild were applied increasingly in recent years (Zeleke et al., 2013). Both 16S rRNA gene and functional genes were applied in this study. It seems 16S rRNA gene was more suitable for routine community composition analysis, however, to get a better resolution of functional groups, the specific primers targeting on the functional genes would be necessary. For example, different ANME clades (referred to as ANME −2a, −2b, −2c, and − 3) were revealed by the *mcr*A gene, while those clades together occupied a relatively lower proportion in the whole archaeal community (ranging from 0.04 to 1.70%) based on the archaeal 16S rRNA gene (Table S3). In addition, the relative abundance of SRB was extremely low in all samples based on the analysis of bacterial 16S rRNA genes. In fact, SRB was known belonging to several diverse clusters, a combination of different primers or probes are needed to cover entire SRB communities specifically (Lücker et al., 2007), and 16S rRNA gene-based monitoring of SRB is particularly not sufficient. Therefore, it is necessary to use specific primers of functional genes combined with 16S rRNA to get a comprehensive picture of the microbial communities, and this is particularly true for the environmental samples containing complex microbial gene pools (Lücker et al., 2007; He et al., 2015).

## Data availability statement

The original contributions presented in the study are publicly available. This data can be found at the NCBI website with accession numbers PRJNA855055, PRJNA855057, PRJNA855285, PRJNA855280.

## Author contributions

QJ designed the study, performed analysis of this study, and wrote the manuscript. HJ designed the study and revised this manuscript. HL and MD revised this manuscript. All authors contributed to the article and approved the submitted version.

## Funding

This study was supported by the Hainan Province Science and Technology special fund (ZDKJ2021036; ZDKJ2019011), the Hainan Provincial Natural Science Foundation of China for High-level Talents (420RC677), the National Natural Science Foundation of China (41776147), and awards from the Senior User Project of RV KEXUE (KEXUE2021GH01).

## Conflict of interest

The authors declare that the research was conducted in the absence of any commercial or financial relationships that could be construed as a potential conflict of interest.

## Publisher’s note

All claims expressed in this article are solely those of the authors and do not necessarily represent those of their affiliated organizations, or those of the publisher, the editors and the reviewers. Any product that may be evaluated in this article, or claim that may be made by its manufacturer, is not guaranteed or endorsed by the publisher.

## Footnotes

^1^ www.novogene.com

^2^ http://huttenhower.sph.harvard.edu/LEfSe/

^3^ http://www.ncbi.nlm.nih.gov
