# Supplementary material for: Biogeographic distributions of microbial communities associated with anaerobic methane oxidation in the surface sediments of deep-sea cold seeps in the South China Sea
Source: Front Microbiol. 2022 Dec 23;13:1060206. doi: 10.3389/fmicb.2022.1060206 (PMC9822730; doi:10.3389/fmicb.2022.1060206)
Supplement: Supplementary file 1 [file Data_Sheet_1.docx]

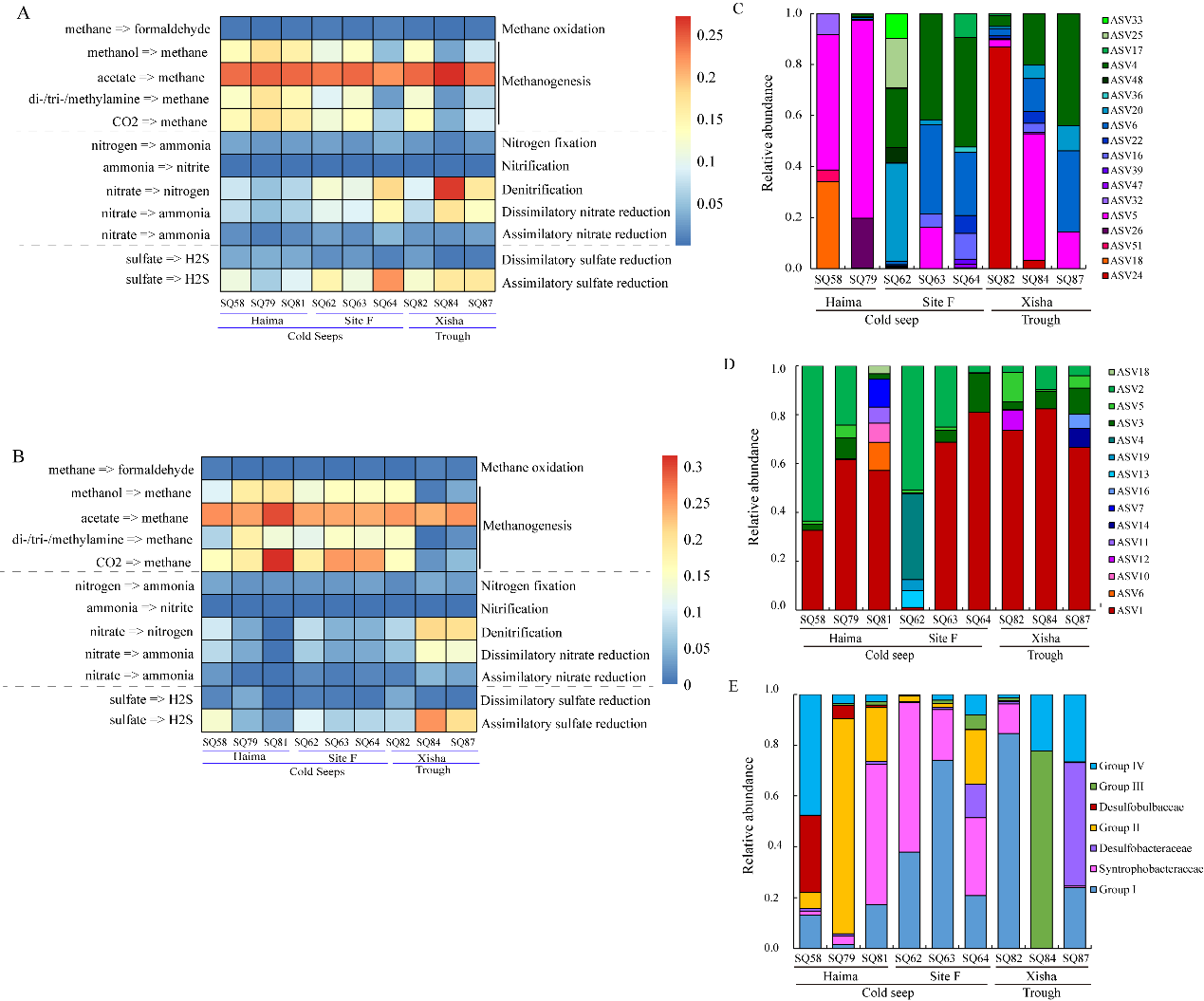


**FIGURE S1.** Heatmaps of major predicted pathways in the methane, nitrogen and sulphur cycles among different regions for bacteria (C) and archaea (D); The community composition of *mcr*A ANME-2d (C), *pmo*A NC10 (D) and *dsr*B (E).

TABLE S1 Primers for PCR and qPCR used in this study.

| Primer | Targeted Microbial Genes | Methods | Sequence（5’-3’） | References |
| --- | --- | --- | --- | --- |
| A189_b F | NC10 *pmo*A | PCR | GGN GAC TGG GAC TTY TGG | (Luesken et al., 2011) |
| cmo682R |  | PCR | AAA YCC GGC RAA GAA CGA |  |
| cmo182F |  | PCR, qPCR | TCA CGT TGA CGC CGA TCC |  |
| cmo568R |  | PCR, qPCR | GCA CAT ACT CCA TCC CCA TC |  |
| McrA169F | ANME-2d *mcr*A | PCR | GCA GCA ATC ACC AAG AAG AGA GG | (Vaksmaa et al., 2017) |
| McrA997F |  | PCR | ATC TGG CTC GGY GGC TAC ATG T |  |
| McrA1360R |  | PCR | TGC CTC TTT GTG GAG GTA CAT GGA |  |
| McrA159F |  | qPCR | AAA GTG CGG AGC AGC AAT CAC C |  |
| McrA345R |  | qPCR | TCG TCC CAT TCC TGC TGC ATT GC |  |
| DSR1F | *dsr*B | PCR | ACS CAC TGG AAG CAC G | (Rampinelli et al., 2008;  Geets et al., 2006) |
| DSRp2060F |  | PCR, qPCR | CAA CAT CGT YCA YAC CCA GGG |  |
| DSR4R |  | PCR, qPCR | GTG TAG CAG TTA CCG CA |  |
| Bac16S_F | 16S rRNA | PCR, qPCR | ACTCCTACGGGAGGCAGCAG | (Liu et al., 2016) |
| Bac16S_R |  | PCR, qPCR | GGACTACHVGGGTWTCTAAT |  |
| Arc16S_F |  | PCR, qPCR | CCCTAYGGGGYGCASCAG | (Takai and Horikoshi, 2000) |
| Arc16S_R |  | PCR, qPCR | GGACTACVSGGGTATCTAAT |  |

TABLE S2 Sequencing information of 16S rRNA and functional genes in this study

| Station | | Archaeal 16S rRNA | | Bacterial 16S rRNA | | *mcr*A | | *pmo*A | | *dsr*B | |
| --- | --- | --- | --- | --- | --- | --- | --- | --- | --- | --- | --- |
|  |  | Quality Reads | ASVs | Quality Reads | ASVs | Quality Reads | ASVs | Quality Reads | ASVs | Quality Reads | ASVs |
| SQ58 | Haima | 703,90 | 625 | 60,501 | 755 | 95,494 | 38 | 15,156 | 13 | 97,368 | 473 |
| SQ79 |  | 57,790 | 1,433 | 29,005 | 1,366 | 109,852 | 29 | 40,903 | 34 | 78,159 | 487 |
| SQ81 |  | 62,552 | 707 | 40,792 | 381 | NA | NA | 98,552 | 35 | 79,595 | 257 |
| SQ62 | Site F | 57,452 | 1,101 | 46,144 | 1,721 | 71,807 | 55 | 62,863 | 14 | 76,429 | 283 |
| SQ63 |  | 69,726 | 813 | 77,640 | 1,690 | 85,921 | 24 | 132,619 | 8 | 64,319 | 99 |
| SQ64 |  | 46,403 | 380 | 26,082 | 1,674 | 100,385 | 29 | 56,533 | 38 | 62,384 | 237 |
| SQ82 | Xisha trough | 67,747 | 688 | 29,577 | 1,605 | 56,718 | 38 | 76,244 | 46 | 51,583 | 203 |
| SQ84 |  | 59,262 | 439 | 81,774 | 714 | 41,898 | 55 | 65,730 | 33 | 110,367 | 28 |
| SQ87 |  | 58,153 | 602 | 68,594 | 1,647 | 76,422 | 37 | 76,126 | 34 | 76,688 | 46 |

TABLE S3 Functional gene-related microbial communities in bacterial and archaeal 16S rRNA genes.

| Taxon | SQ58 | SQ79 | SQ81 | SQ62 | SQ63 | SQ64 | SQ82 | SQ84 | SQ87 |
| --- | --- | --- | --- | --- | --- | --- | --- | --- | --- |
| Desulfobulbus | 28 | 176 | 2 | 0 | 14 | 11 | 5 | 0 | 0 |
| Desulfococcus | 33 | 0 | 0 | 0 | 0 | 0 | 0 | 0 | 0 |
| Desulfobacteraceae | 116 | 153 | 27 | 68 | 133 | 0 | 131 | 0 | 28 |
| Methylomirabilis oxyfera (NC10) | 22 | 58 | 14 | 113 | 441 | 50 | 192 | 4155 | 1360 |
| Methanoperedenaceae ( ANME-2d) | 12 | 0 | 0 | 0 | 0 | 0 | 0 | 0 | 0 |
| ANME-3 | 52 | 0 | 2783 | 0 | 0 | 0 | 0 | 0 | 0 |
| ANME-2a/2b | 44 | 0 | 203 | 2 | 0 | 0 | 0 | 0 | 0 |
| ANME-2c | 32 | 0 | 4 | 0 | 0 | 0 | 0 | 0 | 0 |
